# Expression of concern: Application of 1,4-bis(3-methylimidazolium-1-yl)butane ditribromide [bMImB]·(Br_3_)_2_ ionic liquid reagent for selective oxidation of sulfides to sulfoxides

**DOI:** 10.1039/d4ra90112f

**Published:** 2024-10-01

**Authors:** Abbas Amini Manesh, Fereshteh Hosseini Eshbala, Saba Hemmati, Hojat Veisi

**Affiliations:** a Department of Chemistry, Payame Noor University Tehran Iran a_aminima@yahoo.com +98 81 32542400; b Research Center of Oils and Fats, Kermanshah University of Medical Sciences Kermanshah Iran

## Abstract

Expression of concern for ‘Application of 1,4-bis(3-methylimidazolium-1-yl)butane ditribromide [bMImB]·(Br_3_)_2_ ionic liquid reagent for selective oxidation of sulfides to sulfoxides’ by Abbas Amini Manesh *et al.*, *RSC Adv.*, 2015, **5**, 70265–70270, https://doi.org/10.1039/C5RA06182B.

*RSC Advances* is publishing this expression of concern in order to alert readers that concerns have been raised over the integrity of the data published in this article. The authors have been contacted but have not responded to requests to provide raw data. An expression of concern will continue to be associated with the article until a conclusive outcome is reached.

Signed: Laura Fisher

Date: 16th September 2024

Executive Editor, *RSC Advances*

